# Mechanism of inhibition of Shiga-toxigenic *Escherichia coli* SubAB cytotoxicity by steroids and diacylglycerol analogues

**DOI:** 10.1038/s41420-017-0007-4

**Published:** 2018-02-14

**Authors:** Kinnosuke Yahiro, Sayaka Nagasawa, Kimitoshi Ichimura, Hiroki Takeuchi, Kohei Ogura, Hiroyasu Tsutsuki, Takeshi Shimizu, Sunao Iyoda, Makoto Ohnishi, Hirotaro Iwase, Joel Moss, Masatoshi Noda

**Affiliations:** 1 Department of Molecular Infectiology, Graduate School of Medicine, Chiba University, Chiba, Japan; 2 Department of Legal Medicine, Graduate School of Medicine, Chiba University, Chiba, Japan; 3 Pathogenic Microbe Laboratory, Research Institute, National Center for Global Health and Medicine, Tokyo, Japan; 4 Department of Microbiology, Graduate School of Medical Sciences, Kumamoto University, Kumamoto, Japan; 5 Department of Bacteriology I, National Institute of Infectious Diseases, Tokyo, Japan; 6 Cardiovascular and Pulmonary Branch, National Heart, Lung, and Blood Institute, National Institutes of Health, Bethesda, MD USA

## Abstract

Shiga toxigenic *Escherichia coli* (STEC) are responsible for a worldwide foodborne disease, which is characterized by severe bloody diarrhea and hemolytic uremic syndrome (HUS). Subtilase cytotoxin (SubAB) is a novel AB_5_ toxin, which is produced by Locus for Enterocyte Effacement (LEE)-negative STEC. Cleavage of the BiP protein by SubAB induces endoplasmic reticulum (ER) stress, followed by induction of cytotoxicity in vitro or lethal severe hemorrhagic inflammation in mice. Here we found that steroids and diacylglycerol (DAG) analogues (e.g., bryostatin 1, Ingenol-3-angelate) inhibited SubAB cytotoxicity. In addition, steroid-induced Bcl-xL expression was a key step in the inhibition of SubAB cytotoxicity. Bcl-xL knockdown increased SubAB-induced apoptosis in steroid-treated HeLa cells, whereas SubAB-induced cytotoxicity was suppressed in Bcl-xL overexpressing cells. In contrast, DAG analogues suppressed SubAB activity independent of Bcl-xL expression at early time points. Addition of Shiga toxin 2 (Stx2) with SubAB to cells enhanced cytotoxicity even in the presence of steroids. In contrast, DAG analogues suppressed cytotoxicity seen in the presence of both toxins. Here, we show the mechanism by which steroids and DAG analogues protect cells against SubAB toxin produced by LEE-negative STEC.

## Introduction

Shiga-toxigenic *Escherichia coli* (STEC) infection is an important worldwide cause of human foodborne gastrointestinal diseases^1^. The most popular STEC serotype, O157:H7, produces Shiga toxin 1 (Stx1) and/or Stx2^2^, which cause severe bloody diarrhea, hemorrhagic colitis and hemolytic-uremic syndrome^1^.

A recent epidemiological study showed that Locus for Enterocyte Effacement (LEE)-negative STEC infection increased significantly during the years 2000–2010^3^. One of the LEE-negative STEC strains, STEC O113:H21 strain 98KN2 was responsible for an outbreak of HUS in Australia^4^. This STEC strain produced not only Stx2 but also a novel AB_5_ toxin, subtilase cytotoxin (SubAB). SubAB, which is mainly produced by LEE-negative STEC serotypes^5^, consists of a subtilase-like A subunit (35-kDa) and pentamer of B subunits, which binds to cell surface receptors^4^. After SubAB binds to its surface receptors^6–8^, the toxin translocates into cells through clathrin-mediated^9^ or lipid rafts- and actin-dependent pathways^10^ and then cleaves at a specific site on the chaperone protein BiP/Grp78 in the endoplasmic reticulum (ER)^4^. BiP cleavage by SubAB causes ER stress, followed by activation of ER-stress sensor proteins (e.g., IRE1, ATF6, PERK)^11,12^, which initiate cell damage pathways^11,12^ and various cell responses including inhibition of iNOS synthesis^13^ and stress granule formation^14^. In addition, administration of SubAB to mice causes a lethal severe hemorrhagic inflammation, injury to intestinal cells, extensive microvascular thrombosis, evidence of histological damage in kidneys, and liver, and dramatic splenic atrophy^15–17^.

The clinical treatment of STEC infection is not consistent worldwide. A new approach, steroid pulse therapy has been used as an effective treatment in severe STEC infection^18^. Our recent study showed that the PKC activator, PMA (phorbol 12-myristate 13-acetate), suppressed SubAB-induced PARP cleavage^14^. PMA is a diacylglycerol (DAG) analogue and a potent tumor promoter^19^; other DAG analogues (e.g., bryostatin 1, ingenol-3-angelate) are of clinical interest^20,21^. These analogues have important biological effects, including anti-tumor promoter activity^22,23^, improved status of patients with Alzheimer’s disease^24,25^ and reactivation of latent HIV-1^26^. A previous study showed that DAG and DAG analogues activate the Protein kinase C (PKC) family of proteins, and thereby regulate cell proliferation^20^. They also bind to Ras guanyl nucleotide-releasing proteins (RasGRPs), leading to activation of Ras, and eventually apoptosis^27,28^. Thus, these findings suggest that there may already exist potential therapies for STEC infection that are currently in clinical practice. However, the inhibitory mechanisms of these re-purposed drugs are unknown. Here, we investigated the mechanism in which steroids and DAG analogues inhibit STEC-produced toxin (e.g., SubAB, Stx2)-mediated pathways, leading to cell death.

## Results

### Steroids and DAG analogues inhibit SubAB-induced cell death signaling

**Fig. 1:**
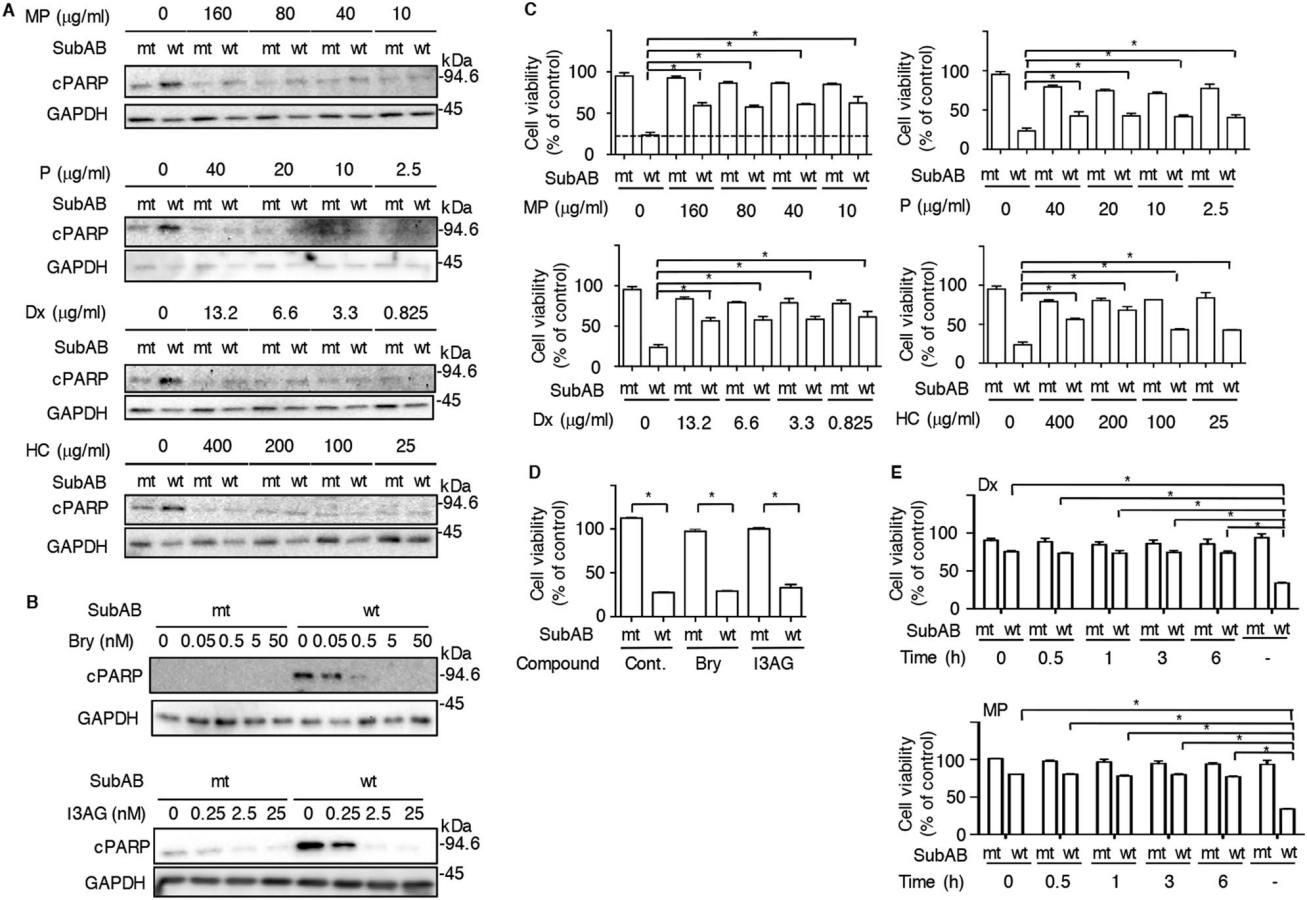
Steroids and DAG analogues inhibit SubAB-induced cytotoxicity **a**, **b** HeLa cells (2.0 × 10^4^/well) were treated for 30 min with the indicated concentrations of methyl prednisolone (MP), dexamethasone (Dx), prednisolone (P), hydroxycortisone (HC), bryostatin 1 (Bry) or ingenol-3-angelate (I3AG), and then incubated for 12 h with mt or wt SubAB (0.2 μg/ml). Cell lysates were subjected to immunoblotting with the indicated antibodies. GAPDH served as a loading control. **c** Cells were treated for 30 min with or without the indicated compounds in a dose-dependent manner, and then incubated for 48 h with mt or wt SubAB (0.2 μg/ml). Cell viability was determined with a Cell Counting Kit. Data are the means ± SD from three separate triplicate experiments. Student’s t-test was utilized for comparisons with SubAB-treated cells without inhibitor. *P < 0.01. **d** Cells were treated for 30 min with or without Bry (10 nM) or I3AG (2.5 nM), and then incubated for 48 h with mt or wt SubAB (0.2 μg/ml). Cell viability was determined with a Cell Counting Kit. Data are the means ± SD from three separate triplicate experiments. Student’s *t*-test was utilized for comparisons with mutant SubAB-treated cells. *P < 0.005. **e** HeLa cells were incubated with mt or wt SubAB (0.2 μg/ml) for the indicated times. Then, Dx (3.3 μg/ml) or MP (40 μg/ml) was added to cells for 48 h. Cell viability was determined with a Cell Counting Kit. Data are the means ± SD from three separate triplicate experiments. Student’s *t*-test was utilized for comparisons with SubAB-treated cells without inhibitor. *P < 0.01

We investigated the effect of steroids (e.g., dexamethasone (Dx), methyl prednisolone (MP), prednisolone (P), hydroxycortisone (HC)) or DAG analogues (e.g., bryostatin1, ingenol-3-angelate) on the SubAB-induced apoptotic pathway in HeLa cells. These compounds are used currently in clinical practice^18,28^. First, cells were incubated with the indicated concentration of drugs in the presence of mutant SubAB (mt) or wild type SubAB (wt), and then PARP cleavage was quantified after 24 h and cell viability was determined after 48 h. SubAB-induced PARP cleavage was inhibited by the steroids at low concentrations (Fig. 1a). Further, SubAB-induced PARP cleavage was suppressed by bryostatin 1 at concentrations < 5 nM and ingenol-3-angelate (I3AG) at concentrations < 2.5 nM (Fig. 1b). Bryostatin 1 alone and I3AG alone at these concentrations did not cause cell damage after a 3 h incubation. After a 48 h incubation, SubAB significantly decreased cell viability, which was reversed in the presence of MP and Dx, but not P, HC, bryostatin 1 or I3AG (Fig. 1c and d). Next, after incubation of HeLa cells with SubAB for the indicated times, we added Dx or MP and measured cell viability after 48 h. The decreased cell viability seen in SubAB-treated cells served as a positive control. Effects of SubAB intoxication were significantly reversed by the presence of Dx and MP even after a 6 h incubation (Fig. 1e). These findings suggested that the steroids (e.g., MP, Dx) suppressed SubAB-induced cell death, while DAG analogues inhibited SubAB-induced cell death signaling at the early time points following intoxication.

### Steroid-induced Bcl-xL expression inhibits cell death by SubAB

**Fig. 2:**
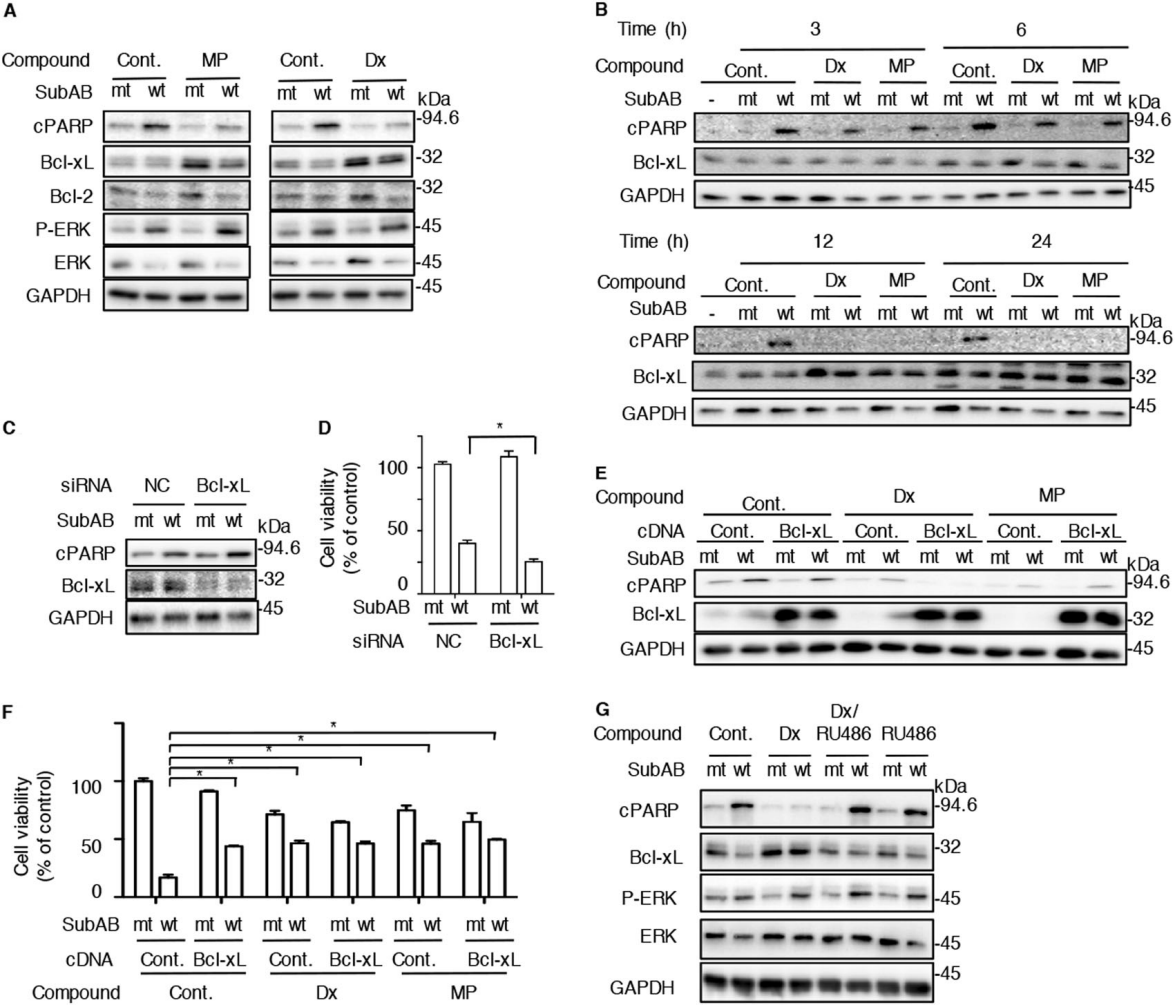
SubAB-induced cytotoxicity is inhibited by steroid-induced Bcl-xL expression **a**, **b** HeLa cells (2.0 × 10^4^/well) were treated with mt or wt SubAB (0.2 μg/ml) in the presence or absence of MP (40 μg/ml) or Dx (3.3 μg/ml) for 12 h **(a)** and for the indicated time points (**b**). Cell lysates were subjected to immunoblotting with the indicated antibodies. GAPDH served as a loading control. **c**, **d** Control or Bcl-xL siRNA-transfected cells were incubated for 3 h **(c)** or 48 h **(d)** with mt or wt SubAB (0.2 μg/ml). Cell lysates were subjected to immunoblotting with the indicated antibodies. Cell viability was determined with a Cell Counting Kit. Data are the means ± SD from three separate triplicate experiments. Student’s t-test was utilized for comparisons with SubAB-treated cells without inhibitor. *P < 0.01. **e**, **f** Control or Bcl-xL plasmid-transfected cells were incubated for 3 h **(e)** or 48 h **(f)** with mt or wt SubAB (0.2 μg/ml). Cell lysates were subjected to immunoblotting with the indicated antibodies. Cell viability was determined with a Cell Counting Kit. Data are the means ± SD from three separate triplicate experiments. Student’s *t*-test was utilized for comparisons with SubAB-treated cells without inhibitor. *P < 0.05. **g** HeLa cells (2.0 × 10^4^/well) were treated for 30 min with Dx (3.3 μg/ml) in the presence or absence of RU486 (10 μM), and then incubated for 12 h with mt or wt SubAB (0.2 μg/ml). Cell lysates were subjected to immunoblotting with the indicated antibodies. GAPDH served as a loading control. Experiments were repeated three times with similar results

Steroids can either enhance or suppress apoptosis^29,30^. Dx inhibits TRAIL-induced apoptosis through up-regulation of anti-apoptotic protein Bcl-xL expression^31^. We investigated the level of Bcl-xL protein. In the presence of MP and Dx, SubAB-induced PARP cleavage was suppressed and the amount of Bcl-xL, but not Bcl-2, was significantly increased. Both MP and Dx treatment enhanced SubAB-induced ERK phosphorylation (Fig. 2a). Next, we examined the time course of Bcl-xL expression with mt or wt SubAB in the presence or absence of MP or Dx. As shown in Fig. 2b, at the time points prior to 6 h, SubAB-induced PARP cleavage was inhibited slightly and Bcl-xL expression was also increased slightly. In contrast, after a 12 h incubation, SubAB-induced PARP cleavage was completely suppressed and Bcl-xL expression in the presence of MP and Dx was dramatically enhanced. To determine whether Bcl-xL was involved in SubAB-induced PARP cleavage, cells treated with Bcl-xL knockdown by specific siRNA were incubated with SubAB (Fig. 2c). After 24 h, SubAB-induced PARP cleavage was promoted in Bcl-xL knockdown cells. The decreased cell viability caused by SubAB was promoted in Bcl-xL knockdown cells compared to control siRNA-transfected cells (Fig. 2d). Furthermore, in Bcl-xL overexpressing cells, SubAB-induced PARP cleavage was suppressed and effects of SubAB on cell viability were reversed, similar to that was seen in Dx- or MP-treated cells (Fig. 2e and f). Next, to determine whether steroid suppression of SubAB-induced apoptosis was mediated by cell signaling through the steroid receptor, we examined the effect of RU486, which acts as a glucocorticoids receptor (GR) antagonist^32^, on SubAB activity in the presence or absence of Dx (Fig. 2g). Although RU486 alone did not affect SubAB activity in HeLa cells, Dx suppression of PARP cleavage by SubAB was not observed in the presence of RU486. In agreement with this result, Dx-induced Bcl-xL expression was not up-regulated in the presence of RU486. However, RU486 did not interfere with Dx-enhanced ERK phosphorylation by SubAB. These findings suggest that steroid-induced Bcl-xL expression was mediated by the steroid receptor, which also protects cells from SubAB activity.

### DAG analogues inhibit SubAB-induced apoptotic pathway via a PKC pathway-mediated Bak/Bax conformational change

**Fig. 3:**
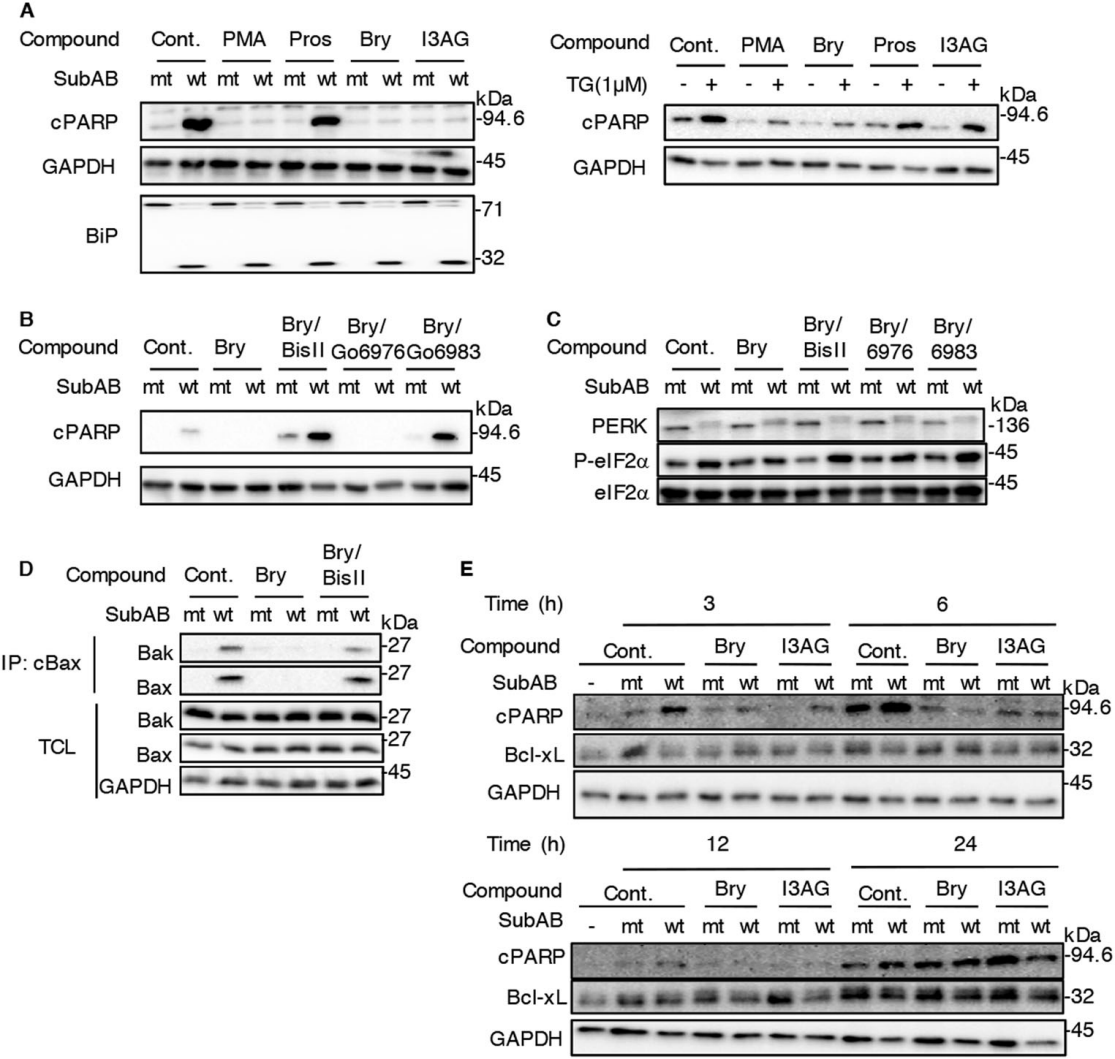
Effects of PKC activators on SubAB-induced PARP cleavage **a** HeLa cells were treated for 30 min with PMA (10 nM), prostratin (10 nM), Bry (10 nM) or I3AG (2.5 nM), and then incubated for 3 h with mt or wt SubAB (0.2 μg/ml) (left panel) or thapsigargin (1 μM) (right panel). Cell lysates were subjected to immunoblotting with the indicated antibodies. **b**, **c** HeLa cells were treated for 30 min with Bry (10 nM) in the presence or absence of 5 μM Bisindolylmaleimide II (Bis II), 5 μM Go6976 or 5 μM Go6983, and then incubated for 3 h with 200 ng/ ml of SubA_S272A_B (mt) or SubAB (wt) (left panel), or Thapsigargin (1 μM) (right panel). Cell lysates were subjected to immunoblotting with the indicated antibodies. **d** Cells were treated for 3 h with the indicated compounds in the presence of mt or wt SubAB (0.2 μg/ml). Cells were lysed and proteins were immunoprecipitated with conformation-specific anti-Bax (cBax) monoclonal antibodies as described in Materials and Methods. The immunocomplexes (IP) or total cell lysates (TCL) were analyzed by SDS-PAGE, followed by immunoblotting with anti-Bax and anti-Bak antibodies. GAPDH served as a loading control. **e** HeLa cells were treated for the indicated times with or without Bry (10 nM) or I3AG (2.5 nM) in the presence of mt or wt SubAB (0.2 μg/ml). Cell lysates were subjected to immunoblotting with the indicated antibodies. All experiments were repeated three times with similar results

As shown in Fig. 3a, PMA, bryostatin 1 and I3AG, but not prostratin, suppressed SubAB-induced PARP cleavage. Both bryostatin 1 and I3AG treatment did not inhibit BiP cleavage by SubAB. In contrast, PARP cleavage induced by a general ER-stress inducer thapsigargin (TG) was suppressed by PMA and bryostatin 1, but not by I3AG and prostratin (Fig. 3a). Thus, these results show that bryostatin 1 inhibits SubAB activity and that it acts by a different pathway from TG. Next, we examined the involvement of PKC activity in bryostatin 1- or I3AG-suppression of PARP cleavage by SubAB, using PKC-pathway blockers (e.g., Gö6976, Bis II, Gö6983) (Fig. 3b). Treatment of HeLa cells with Gö6976, which inhibits PKCα and PKCβ isoforms, did not affect bryostatin 1 activity. In the presence of Bis II and Gö6983, bryostatin 1- or I3AG-suppression of PARP cleavage by SubAB was not observed (Fig. 3b).

We determined the process by which the SubAB-induced apoptotic pathway is inhibited by bryostatin 1 and I3AG. As shown in Fig. 3c, compared to control cells, SubAB-induced PERK phosphorylation and eIF2α phosphorylation were not changed either with or without bryostatin 1 and I3AG in the presence of PKC-pathway blockers. Next, we examined the effect of bryostatin 1 and I3AG on SubAB-induced Bax/Bak conformational changes in the presence or absence of PKC-pathway blocker Bis II. Our results demonstrated that both bryostatin 1 and I3AG suppressed SubAB-induced Bax/Bak conformational changes. In agreement with the results of the PARP cleavage study shown in Fig. 3b, treatment of HeLa cells with Bis II inhibited SubAB-induced Bax/Bak conformational changes even in the presence of bryostatin 1 and I3AG (Fig. 3d). Thus, these data suggest that the inhibitory effect of bryostatin 1 and I3AG on SubAB-induced apoptosis might require a PKC-dependent pathway. We next monitored SubAB-induced PARP cleavage and Bcl-xL expression in the presence or absence of bryostatin 1 or I3AG for an extended incubation (Fig. 3e). Treatment cells with bryostatin 1 or I3AG did not affect Bcl-xL expression. During an up to 12 h incubation, SubAB-induced PARP cleavage was suppressed by bryostatin 1 or I3AG. After a 24 h incubation, PARP cleavage was, however, increased with bryostatin 1 or I3AG in the presence of mutant SubAB, suggesting that these compounds have a cytotoxic effect following a long incubation time.

### DAG analogues-activated ERK is a key event in suppression of SubAB-induced apoptosis

**Fig. 4:**
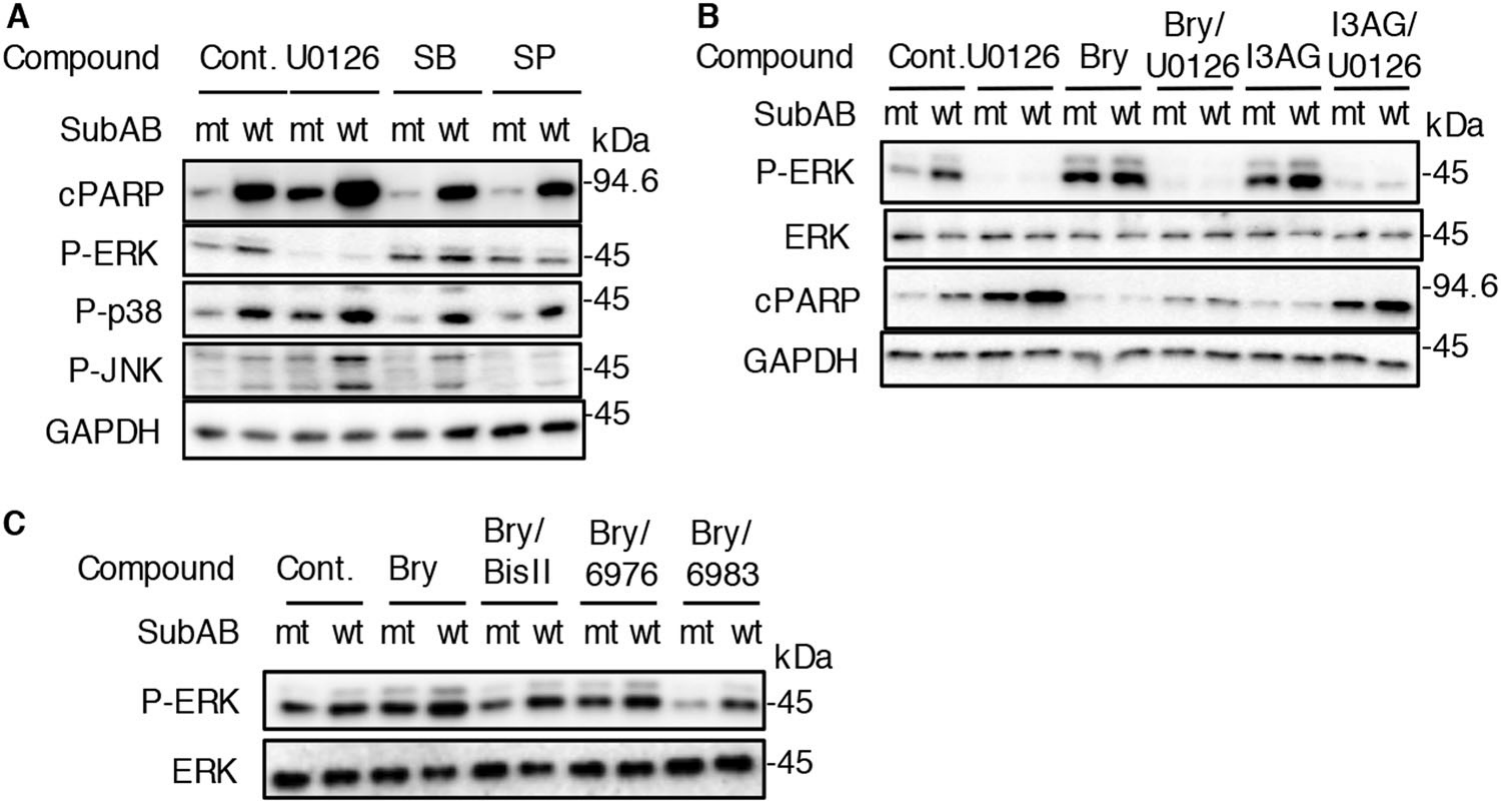
SubAB-induced cell death is regulated by ERK, which is downstream of PKC **a** HeLa cells were treated for 3 h with U0126 (10 μM), SB23590 (20 μM), SP600125 (10 μM) or DMSO as a control in the presence of mt or wt SubAB (0.2 μg/ml). Cell lysates were subjected to immunoblotting with the indicated antibodies. **b** Cells were treated for 30 min with or without Bry (10 nM) or I3AG (2.5 nM) in the presence or absence of U0126 (10 μM), and then incubated for 3 h with mt or wt SubAB (0.2 μg/ml). Cell lysates were subjected to immunoblotting with the indicated antibodies. **c** Cells were treated for 30 min with or without Bry (10 nM) in the presence or absence of 10 μM PKC inhibitors (e.g., Bis II, Go6973, Go6986), and then incubated for 3 h with mt or wt SubAB (0.2 μg/ml). Cell lysates were subjected to immunoblotting with anti-phospho-ERK (p-ERK) or anti-ERK antibodies. All experiments were repeated three times with similar results

It is known that activation of ERK pathway plays a functional role in protection from apoptosis^33^. Inhibition of ERK increased SubAB-induced apoptosis in rat tubular epithelial cell line NRK-52E^34^. In agreement with this report, ERK inhibitor U0126, but not JNK inhibitor SP600125 and p38 inhibitor SB23590, enhanced SubAB-induced PARP cleavage in HeLa cells (Fig. 4a). We next investigated the effect of ERK activation on SubAB activity suppressed by DAG analogues. Treatment of HeLa cells with bryostatin 1 or I3AG increased ERK phosphorylation and suppressed PARP cleavage by SubAB. In the presence of U0126, DAG analogues-induced ERK phosphorylation was completely inhibited and DAG analogues-suppressed PARP cleavage by SubAB was canceled (Fig. 4b). Next, we examined whether PKC regulates ERK activation. As shown in Fig. 4c, PKC inhibitors (e.g., Bis II, Go6976, Go6983) did not suppress DAG analogues-induced ERK phosphorylation. These findings indicate that the protective effect of DAG analogues involves ERK activation, which may be upstream of PKCs.

### ERK inhibitor suppresses steroid-induced Bcl-xL expression

**Fig. 5:**
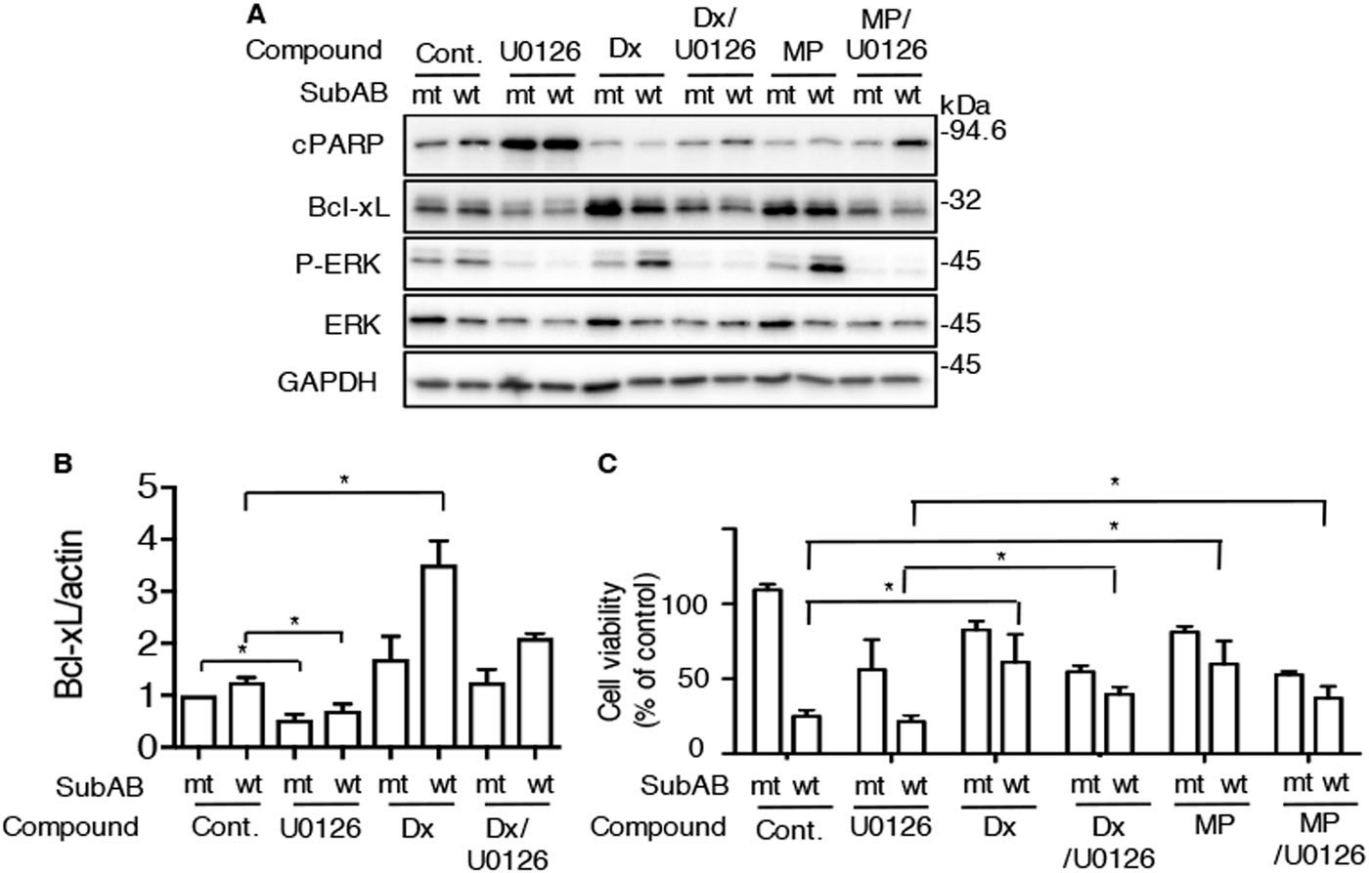
ERK inhibitor suppresses steroid-induced Bcl-xL expression **a** Cells were treated for 30 min with or without Dx (3.3 μg/ml) or MP (40 μg/ml) in the presence or absence of U0126 (10 μM), and then incubated for 12 h with mt or wt SubAB (0.2 μg/ml). Cell lysates were subjected to immunoblotting with the indicated antibodies. **b** Cells were treated as described in above and then incubated for 6 h with the toxins. Total RNA was extracted as described in Materials and Methods. The expression level of Bcl-xL mRNA was measured by real-time qPCR. Data are shown as mean ± SD of values from three independent experiments. Results are shown as fold increase of β-actin as an internal control. **c** Cells were treated for 48 h with the indicated compounds in the presence of mt or wt SubAB (0.2 μg/ml). Cell viability was determined with a Cell Counting Kit. Data are the means ± SD from three separate triplicate experiments. Student’s *t*-test was utilized for comparisons with SubAB-treated cells without inhibitor. *P < 0.05

As described above, steroid-increased Bcl-xL suppressed SubAB-induced cell death. We investigated if ERK activity controls Bcl-xL expression by steroids. After a 24 h incubation, SubAB-induced ERK phosphorylation was increased in the presence of Dx and MP compared to control cells. ERK inhibitor U0126 suppressed Dx- and MP-induced Bcl-xL expression and consequently PARP cleavage by SubAB was observed again (Fig. 5a). Next, to investigate the effect of U0126 and/or Dx on Bcl-xL mRNA level, cells were treated with SubAB in the presence or absence of Dx or U0126. Treatment of HeLa cells with U0126 significantly decreased the expression of Bcl-xL mRNA. Dx-increased Bcl-xL mRNA was reduced by treatment with U0126 (Fig. 5b). In agreement with results in Fig. 5a and  b, steroids suppressed cytotoxicity by SubAB. Treatment of the cells with U0126 increased cytotoxicity by SubAB and interfered with cell protection by Dx or MP (Fig. 5c). Thus, ERK is involved in the regulation of Bcl-xL expression by steroids and also plays an important role in apoptotic signaling induced by SubAB.

### Cytoprotective effect of steroid is decreased by addition of both SubAB and Stx

**Fig. 6:**
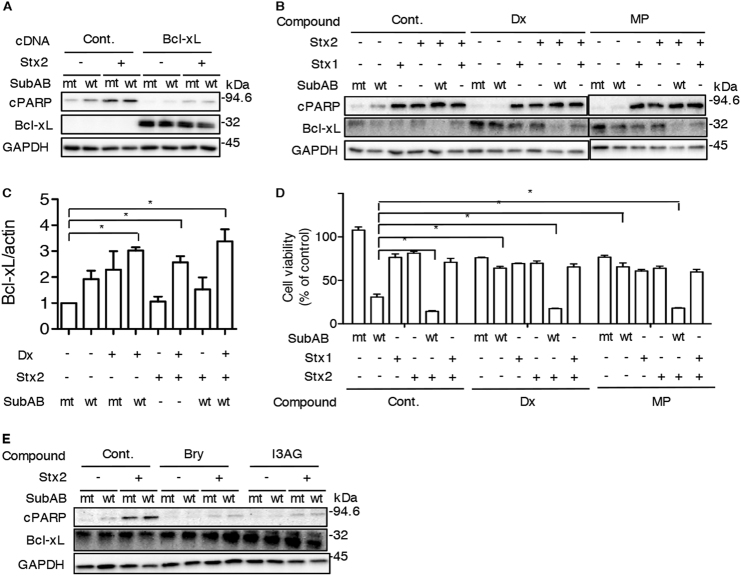
Effects of steroids and DAG analogues on Stxs- and SubAB-induced apoptosis **a** Control- or Bcl-xL-overexpressing cells were incubated for 24 h with mt or wt SubAB (0.2 μg/ml) in the presence or absence of Stx2 (100 ng/ml). Cell lysates were subjected to immunoblotting with the indicated antibodies. **b** Cells were treated for 30 min with or without Dx (3.3 μg/ml) or MP (40 μg/ml), and then incubated for 24 h with mt or wt SubAB (0.2 μg/ml) in the presence or absence of 100 ng/ml Stx1 or Stx2. Cell lysates were subjected to immunoblotting with the indicated antibodies. **c** Cells were treated for 6 h with or without Dx in the presence or absence of the toxins, as described in **b**. Total RNA was extracted as described in Materials and Methods. The expression level of Bcl-xL mRNA was measured by real-time qPCR. Data are shown as mean ± SD of values from three independent experiments. Results are shown as fold increase of β-actin as an internal control. *P < 0.05 **d** Cells were incubated for 48 h with the indicated compounds in the presence of absence of the toxins. Cell viability was determined with a cell counting kit. **e** Cells were incubated for 3 h with Bry (10 nM) or I3AG (2.5 nM) in the presence of the toxins. Cell lysates were subjected to immunoblotting with the indicated antibodies. All experiments were repeated three times with similar results. Data are the means ± SD from three separate duplicate experiments. Student's t-test was utilized for comparisons with SubAB-treated cells without inhibitor. *P < 0.05

Most *subAB*-positive STEC strains harbor the *stx2* gene^35^. We investigated the effect of steroids on both of SubAB- and Stx2-induced cytotoxicity. We first examined PARP cleavage induced by both toxins in Bcl-xL overexpressing cells. In control cells, SubAB-induced PARP cleavage was enhanced by addition of Stx2. PARP cleavage by SubAB and/or Stx2 was attenuated in Bcl-xL overexpressing cells (Fig. 6a). Next, we tested whether steroids protect from toxins-induced apoptosis. SubAB-induced PARP cleavage was enhanced by Stx2, which is in agreement with a previous report^36^. Although Dx or MP treatment inhibited SubAB-induced PARP cleavage, Stx2-, Stx2/SubAB-, and Stx2/Stx1-induced PARP cleavage was not suppressed. Dx- or MP-stimulated Bcl-xL expression was dramatically decreased by treatment with Stx2/SubAB (Fig. 6b). We further assessed whether these toxins affect Bcl-xL transcription in the presence of Dx by real-time qPCR. The levels of Bcl-xL mRNA were increased when cells were incubated with wild-type toxins and Dx (Fig. 6c). Treatment of cells with SubAB/Stx2 did not suppress Dx-induced Bcl-xL mRNA expression, although the amount of Bcl-xL was decreased by SubAB/Stx2. We next investigated the effect of steroids on SubAB/Stx-induced cytotoxicity (Fig. 6d). Treatment of cells with SubAB/Stx2 enhanced cytotoxicity compared to either SubAB or Stx2 alone. Suppression of SubAB cytotoxicity seen with Dx or MP was not reversed by Stx2. We next tested the effect of DAG analogues on PARP cleavage by SubAB/Stx2. These compounds have inhibitory effects against SubAB- and/or Stx2-induced PARP cleavage (Fig. 6e).

### Effect of steroids and DAG analogues on SubAB-induced PARP cleavage in mouse intestinal organoids and MEF cells

**Fig. 7:**
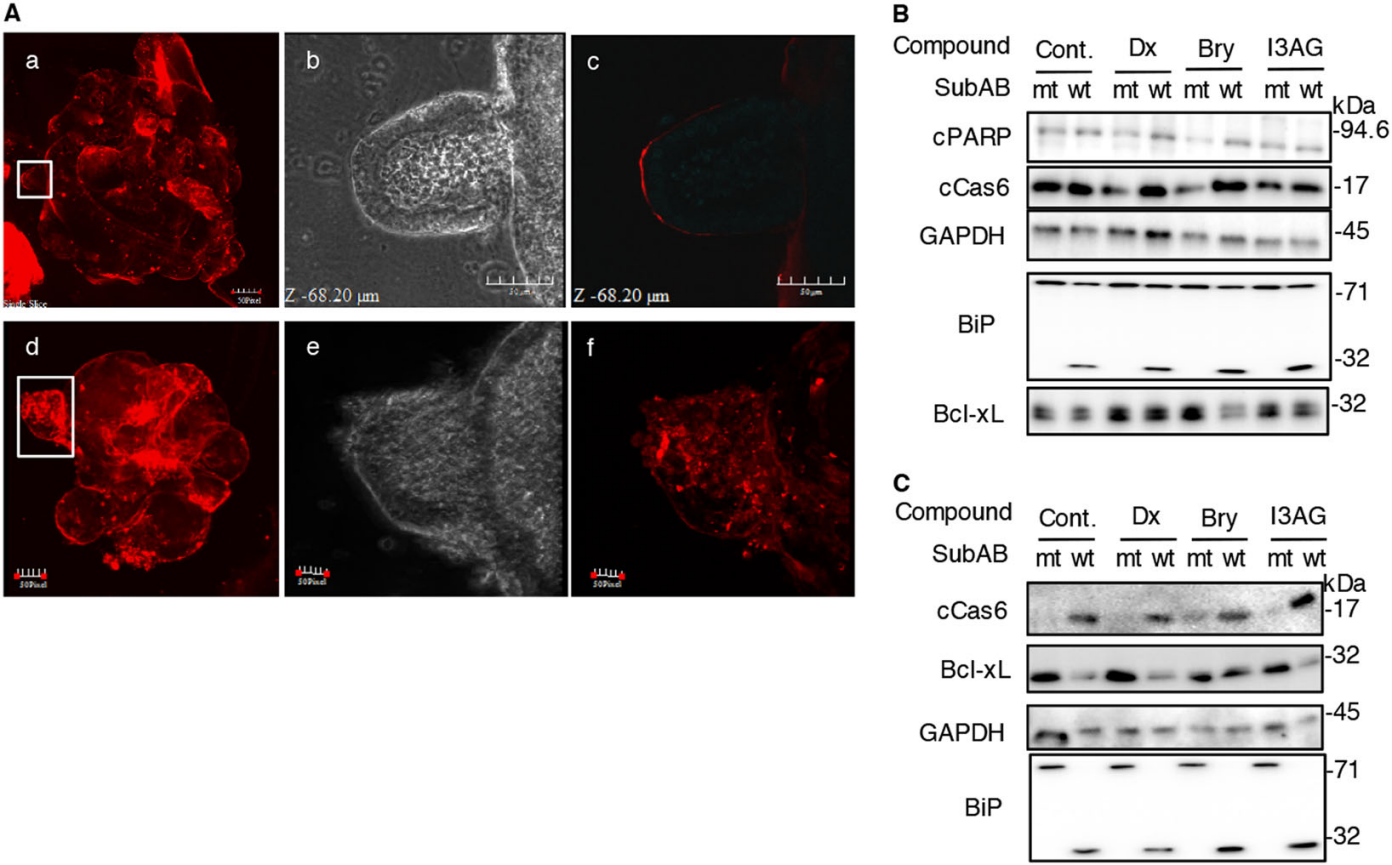
Steroids and DAG analogues did not inhibit SubAB-induced apoptosis in mouse intestinal organoids and MEF cells **a** Mouse intestinal organoids were incubated for 30 min on ice (Top panel) or for 2 h at 37 °C (bottom panel) with cy3-labeled SubAB and then fixed as described in Materials and methods. b, c, e, and f shows high-magnification views of the white rectangular areas in a and d, respectively. Experiments were repeated three times with similar results. **b** The organoids were treated for 12 h with or without Dx (3.3 μg/ml), Bry (10 nM) or I3AG (2.5 nM), and then incubated for 24 h with mt or wt SubAB (0.2 μg/ml). Cell lysates were subjected to immunoblotting with the indicated antibodies. Experiments were repeated three times with similar results. **c** MEF cells were treated for 12 h with or without Dx (3.3 μg/ml), Bry (10 nM) or I3AG (2.5 nM), and then incubated for 24 h with mt or wt SubAB (0.2 μg/ml). Cell lysates were subjected to immunoblotting with the indicated antibodies. Experiments were repeated three times with similar results

First, we investigated if SubAB bound and entered mouse intestinal organoids using cy3-labeled SubAB. Organoids incubated with cy3-SubAB on ice showed SubAB bound to their top (Fig. 7a). Most of cy3-SubAB was attached on the surface and some cy3-SubAB entered into organoids after 2 h incubation at 37 °C (Fig. 7a; d, e, and f). Next, we examined whether Dx or DAG analogues inhibited SubAB-induced PARP cleavage in mouse intestinal organoids and MEF cells. Dx or DAG analogues-treated mouse intestine organoids (Fig. 7b) or MEF cells (Fig. 7c) were incubated for 24 h with mt or wt SubAB. SubAB-induced PARP cleavage or caspase 6 activation was not inhibited in the presence of Dx, bryostatin 1 or I3AG. Dx-enhanced Bcl-xL expression was significantly decreased by SubAB. Our findings suggested that steroids and DAG analogues did not have an inhibitory effect on SubAB activity in mouse cells.

## Discussion

In this study, we observed that steroids or DAG analogues, which have been used in clinical medicine^37^ or clinical trials^24^, have a suppressive effect on SubAB- or Stx-induced cytotoxicity. Stx is a major virulence factor that is responsible for severe gastrointestinal diseases caused by both LEE-positive and -negative STEC infections. In contrast, during LEE-negative STEC infection, SubAB is produced and this toxin causes severe hemorrhagic inflammation and intestinal cell damage^4,15,16^. Although both of these toxins inhibit protein synthesis, their targets are a quite different. Thus, after uptake into cells, Stx causes cytotoxicity through its RNA-*N*-glycosidase activity^1^. In contrast, SubAB is translocated into the ER, cleaves chaperone protein BiP, resulting in ER stress-induced apoptosis^38^. A number of studies have tried to identify Stxs inhibitors to reduce STEC-induced severe damage^39^. Most of these compounds were small molecules, which target the Stx active site, binding sites, intracellular trafficking, or pathways involved in pro-inflammatory or pro-apoptotic signaling, and that partially or completely protected cells from Stx cytotoxicity^40,41^. It has been reported that SubAB-induced PARP cleavage was inhibited by PKC activator PMA^14^ or by a general caspase inhibitor (Z-VAD)^12^.

Recent studies showed that steroid pulse therapy was an effective treatment for severe STEC infection^18,37^. However, it remains unknown why steroids improve severe damage caused by STEC infection. We showed here that steroid-induced Bcl-xL expression suppresses SubAB-induced apoptosis. Non-steroidal anti-inflammatory drugs (NSAIDs) did not affect SubAB-induced apoptotic cell death (Supplementary Fig. 1). Bcl-xL showed an anti-apoptotic ability through formation of a heterodimer with Bax, an apoptotic protein^42^. Previous studies showed that Bcl-xL inhibits cell death under various stresses (e.g., *Clostridium difficile* Toxin A, axotomy-induced neuronal apoptosis, chemotherapeutic drugs)^43–45^. Several transcription factors (e.g., NF-κB, GATA, Ets, PAX3, POU, STATs, c-Myb, HIF-1α) regulate Bcl-xL gene expression^46^. Yu and their colleagues^47^ showed that, in the interleukin 3-dependent hematopoietic cell line Ba/F3, MAPK-dependent GATA-1 phosphorylation leads to transactivation of the E4bp4 gene, Bcl-xL expression and cell proliferation. Petrella et al.^31^ reported that Dx suppressed TRAIL-induced apoptosis by increasing Bcl-xL expression in thyroid cancer cells. In their study, Bcl-xL knockdown decreased cell survival and Bcl-xL overexpression inhibited TRAIL-induced apoptosis. Further, during fibrosarcoma development, Dx-activated glucocorticoid receptor (GR) enhanced Bcl-xL expression, leading to anti-apoptotic effects^48^. Similar to these findings, SubAB-induced apoptosis was inhibited in Bcl-xL overexpressing cells and increased in Bcl-xL knockdown cells. However, Stxs-induced apoptotic signaling was not suppressed in Dx- or MP-treated cells. Steroid-induced Bcl-xL expression was dramatically decreased by SubAB/Stx2, in parallel with sensitization to apoptosis with PARP cleavage as well as decreased cell viability. Further, SubAB/Stx2 did not inhibit Dx-increased Bcl-xL mRNA expression. These results suggest that decreased Bcl-xL expression by SubAB/Stx2 is involved in inhibition of protein synthesis or promotion of protein degradation, but not in transcription.

We did not observe the inhibitory effects of steroids or DAG analogues on SubAB activity in mouse intestinal organoids and MEF cells, although steroids enhanced Bcl-xL expression. These findings suggest that steroid- or DAG analogues-stimulated signal pathways alone is not sufficient for inhibition of SubAB-induced cell death in mouse intestinal organoids and MEF cells. Thus, our data suggest that results with mouse cells do not necessarily apply to human cells.

PKC activators or steroids regulate ERK phosphorylation, which is associated with apoptosis. ERK inhibitor, U0126, enhanced PARP cleavage and decreased cell viability by suppression of Bcl-xL expression. ERK is a member of mitogen-activated protein kinases (MAPKs)^49^. Activation of ERK by phosphorylation has been demonstrated to protect cells from various death signals^50^. Inhibition of ERK activities caused a down-regulation of Bcl-xL expression and induced apoptotic cell death^33^. The difference between mouse and human cells may result in differential activation of ERK. We did not observe DAG analogues-induced ERK activation in mouse intestinal organoid (data not shown). Thus, this may be a reason why DAG analogues did not inhibit SubAB-induced cell death in mouse cells. While the mechanism by which steroids cause ERK activation remains unknown, a previous study demonstrated in rat brain that Dx protects from neonatal hypoxic-ischemic brain injury. It was proposed that Dx interacts with the glucocorticoids receptor (GR), activates the lipocalin-type prostaglandin D synthase (L-PGDS)-induced prostaglandin D2 (PGD2)-DP1 receptor, and upregulates ERK phosphorylation^51^. On the other hand, in lung cancer cell lines, Dx suppressed cell proliferation and cell cycle via the GR-dependent pathway and inhibition of ERK/MAPK signaling^52^. These findings suggest that the effect of steroid-activated ERK is different in various cell types or tissues. In the case of HeLa cells, steroid treatment did not induce ERK phosphorylation, which was enhanced by incubation with SubAB. These findings indicated that steroid-induced Bcl-xL expression is independent of ERK activation. Treatment of HeLa cells with GR antagonist RU486 or ERK inhibitor U0126 inhibited steroid-suppressed PARP cleavage by SubAB. Dx/SubAB-induced ERK phosphorylation was not altered in the presence of RU486. Thus, steroid-increased Bcl-xL expression is essential for GR-mediated cell signaling, which is blocked by inhibition of ERK.

DAG analogues (bryostatin 1 and I3AG) have unique biological activities in cancer chemotherapy^22,23^, treatment of Alzheimer’s disease^24^ or as a drug for promoting HIV eradication^25^. We demonstrated that these compounds dramatically suppress apoptotic activity by SubAB during a 12 h incubation. Bryostatin 1, but not I3AG, inhibited thapsigargin-induced PARP cleavage after a 3 h incubation, suggesting that ER stress signaling by SubAB is different from that of thapsigargin. On the other hand, these compounds increased cytotoxicity in HeLa cells after a 24 h incubation. In addition to prostratin, 1α,25-dihydroxyvitamin D3 (1,25-D3) is also known as a PKC activator in Caco-2 cells^53^, but this compound did not inhibit apoptotic activity by SubAB (data not shown). Thus, some PKC activators have potent ability to suppress toxin activity. Modification of bryostatin 1 or I3AG of structure and their cytotoxic effects may generate compounds working through PKC activation, which may prove to be useful drugs to treat STEC infection.

## Materials and methods

### Reagents

Anti-Bak, anti-BiP/Grp78, anti-Bax, and anti-ERK monoclonal antibodies were from BD Bioscience; anti-PERK, anti-Bcl-xL, anti-Bim, anti-Bak, anti-Bax, anti-eIF2α, anti-phospho-eIF2α, anti-phospho-ERK, anti-cleaved caspase 6 (cCas6) and anti-cleaved poly(ADP-ribose) polymerase (cPARP) antibodies were from Cell Signaling Technology; and anti-GAPDH antibodies were from GeneTex. Anti-SubAB antibodies were prepared as previously described^6^. PKC inhibitor Gö6976 and ERK inhibitor U0126 were obtained from LC Laboratories. SB23590 was from WAKO. PKC activators (PMA and prostratin), Thapsigargin, and PKC inhibitor Gö6983 were from Sigma Aldrich. Bryostatin 1 was from Santa Cruz Biotechnology. PKC inhibitor Bisindolylmaleimide II (Bis II) was from ALEXIS Biochemicals. PKC activator Ingenol-3-angelate (I3AG), SP600125, and steroid antagonist RU486 were from Cayman Chemical. Dexamethasone sodium phosphate (Dx) was from FujiPhama. Prednisolone (P) was from SHIONOGI & CO., Ltd. Methylprednisolone Succinate Na (MP) was from Sawai Pharmaceutical Co., Ltd. Hydrocortisone (HC) was from Taisho Pharmaceutical Co., Ltd.

### Preparation of SubAB and Stx

Recombinant His-tagged SubAB and catalytically inactive mutant SubA_S272A_B were purified by Ni-NTA Agarose (QIAGEN) chromatography as reported previously^54^. According to previous reports, Stx1 was purified by affinity chromatography from lysates of *E. coli* MC1061 containing pKTY1 expression plasmid with *stx1* insert^55^ and Stx2 was purified by column chromatography from lysates of *E. coli* BL21 containing p2AKH-2 expression plasmid with *stx2* insert^56^.

### Cell culture and gene transfection

HeLa cells were cultured at 37 °C in a humidified 5% CO_2_ atmosphere in Eagle’s Minimum Essential Medium (EMEM) (Sigma) containing 10% heat-inactivated fetal bovine serum (FBS), 100 U/ml penicillin and 0.1 mg/ml streptomycin. Mouse embryonic fibroblasts (MEF) were cultured at 37 °C in a humidified 5% CO_2_ atmosphere in Dulbecco’s Modified Eagle’s medium (DMEM) (Sigma) containing 10% heat-inactivated fetal bovine serum (FBS), 100 U/ml penicillin and 0.1 mg/ml streptomycin. Human Bcl-xL expression plasmid was prepared, as described in ref.^57^. Cells were cultured in a 24-well plate (1 × 10^5^/well) overnight and transfected with 0.5 μg of plasmids using Polyethylenimine “Max” (Polysciences. Inc). After 24 h incubation, cells were treated with the toxins for the indicated times. Bcl-xL siRNA (5′- GGGACAGCAUAUCAGAGCU-3′) was designed and validated, as described in ref.^58^. Negative-control siRNAs were purchased from Sigma Aldrich. Cells were transfected with 100 nM of the indicated siRNAs for 48–72 h using Lipofectamine^TM^ RNAiMax transfection reagent (Invitrogen) according to the manufacturer’s protocol. Knockdown of Bcl-xL was confirmed by immunoblotting with the antibodies.

### Mouse intestinal epithelial organoids culture

Animal experiments were approved by Chiba University Animal Welfare committee. For studies involving small intestine, three weeks old BALB/c mice (Japan SLC, Inc.) were used. Mouse intestinal epithelial organoids were prepared according to the manufacturer’s protocol (STEMCELL Technologies). Briefly, the small intestine of mice was excised, washed in cold PBS, and opened lengthwise. After washing with cold PBS, the small intestine was cut into 2 mm pieces, which were washed with cold PBS 15 times until the supernatant was clear. The tissue pieces were suspended at room temperature for 20 min in General Cell Dissociation Reagents (STEMCELL Technologies), then resuspended in cold PBS containing 0.1% BSA (Sigma Aldrich) and filtered through a 70 μm cell strainer (BD Biosciences) into a 50 ml Falcon tube. This step was repeated four times. After centrifugation at 290 × g for 5 min, the supernatant was removed. The precipitated cells were suspended in cold DMEM/F12 medium (STEMCELL Technologies), and the number of crypts were counted using a hemacytometer. After centrifuged at 290 × *g* for 5 min, the pellet was suspended at room temperature in 150 μl of complete IntestiCult^TM^ Organoid Growth Medium (STEMCELL Technologies) containing 100 μg/ml Penicillin/Streptomycin (Sigma Aldrich), Matrigel Matrix (Corning) (150 μl) was added and then the pellet was resuspended, avoiding air bubbles. Matrigel mixture (50 μl) was added into the center of each well of a prewarmed 24-well plate, which was incubated at 37 °C to set the Matrigel. Complete IntestiCult^TM^ Organoid Growth Medium (0.5 ml) was added to each well to cover the Matrigel matrix. The cultures were incubated at 37 °C in 5% CO_2_, with medium changed every second day.

### Cell viability assay

HeLa cells (1 × 10^4^ cells/96-well plate) were treated with the indicated compounds for 30 min, then incubated for 48 h with wild-type (wt) or catalytically inactive mutant (mt) SubAB (200 ng/ml). Cell viability was measured by a Cell Counting Kit (Dojindo) according to the manufacturer’s protocol.

### Immunoprecipitation

Co-immunoprecipitation of conformationally changed Bax or Bak was performed as described previously^12^. Briefly, HeLa cells (3 × 10^5^ cells/12-well plate) were treated with mt or wt SubAB for 3 h. After washing with ice-cold PBS, cells were solubilized with lysis buffer (10 mM HEPES, 150 mM NaCl, 1.5 mM MgCl_2_, 1 mM EGTA, 2% CHAPS, pH 7.4) and incubated for 30 min on ice. After centrifugation at 17,400 × *g* for 15 min at 4 °C, solubilized extracts (100 μg/200 μl) were collected and incubated with conformation-specific anti-Bax antibody (clone 3) (BD Bioscience) at 4 °C for 3 h. Immunoprecipitated complexes were collected by incubation with protein G-Sepharose (Invitrogen) for 1 h, followed by centrifugation for 1 min at 4 °C. After washing the immunocomplexes with lysis buffer three times, proteins were dissolved in 1×SDS-sample buffer, subjected to SDS–PAGE in 15% gels, and transferred to PVDF membranes, which were then visualized by Western blotting using anti-Bax or anti-Bak antibodies (Cell Signaling).

### Immunoblotting analysis

Whole-cell lysates were prepared with 1×SDS-sample buffer, and then heated at 100 °C for 10 min before proteins were analyzed by SDS-PAGE. Separated proteins were transferred to PVDF membranes (Millipore) at 100 V for 1 h, blocked with 5% non-fat milk (Wako) in TBS-T (20 mM Tris-HCl pH 7.6, 137 mM NaCl, 0.1% Tween 20) for 30 min and then incubated with the primary antibodies overnight at 4 °C. After washing with TBS-T, membranes were incubated with horseradish peroxidase-labeled secondary antibodies for 1 h at room temperature. Bands were detected by EzWestLumi One (ATTO corporation) using LAS-1000 (Fuji Film).

### Real-time quantitative PCR analysis

Total RNA from HeLa cells (2 × 10^5^ cells) was extracted by ISOGEN II (WAKO) as described in the instruction manual. First-strand cDNA synthesis was performed with a PrimeScript™ II 1st strand cDNA Synthesis Kit (Takara Bio). Real-time quantitative PCR (qPCR) analysis was conducted using KOD SYBR qPCR Mix (TOYOBO) with the fluorescent dye SYBR Green and a ABI Prism 7000 (PerkinElmer Life Sciences) for detection. Primer pairs for the Bcl-xL gene PCR were synthesized according to the previous report^59^. The PCR protocol was performed as described in the instruction manual. The reaction mixture was activated at 98 °C for 2 min of 1 cycle, followed by denaturation for 10 s at 98 °C, annealing for 10 secs at 60 °C and extension for 30 s at 68 °C, for 45 cycles. The dissociation curve for each sample was analyzed to verify the specificity of each reaction. The relative mRNA expression levels of Bcl-xL genes were determined by the delta-delta Ct method and normalized to β-actin expression. Specific primers were as follows: sense; 5′-TTCTACAATGAGCTGCGTGTG-3′ and antisense 5′-GGGGTGTTGAAGGTCTCAAA-3′.

### Confocal microscopy analysis

Mouse intestinal organoids were treated with Cy3-labeled SubAB in IntestiCult^TM^ Organoid Growth Medium for 30 min on ice, washed with cold PBS and then fixed on coverslips using Smear Gell (GenoStaff Inc. Japan).

Cy3-labeled SubAB treated mouse intestinal organoids were incubated at 37 °C for 2 h on Matrigel matrix-coated coverslips, and then fixed with 4% paraformaldehyde for 1 h. Cells on the coverslips were mounted on glass slides using ProLong Gold antifade reagent with DAPI (Invitrogen). The stained cells were visualized using a FV10i-LIV confocal microscope (Olympus). The images were arranged with Adobe Photoshop Elements 15.

### Statistical analysis

Student’s *t*-test was used to determine significant difference when only two treatment groups were being compared.

## Electronic supplementary material

Figure S1

DECLARATION OF CONTRIBUTIONS TO ARTICLE
