# Supplementary figures and images for: Mechanism of inhibition of Shiga-toxigenic Escherichia coli SubAB cytotoxicity by steroids and diacylglycerol analogues
Source: Cell Death Discov. 2018 Feb 14;4:22. doi: 10.1038/s41420-017-0007-4 (PMC5841432; doi:10.1038/s41420-017-0007-4)

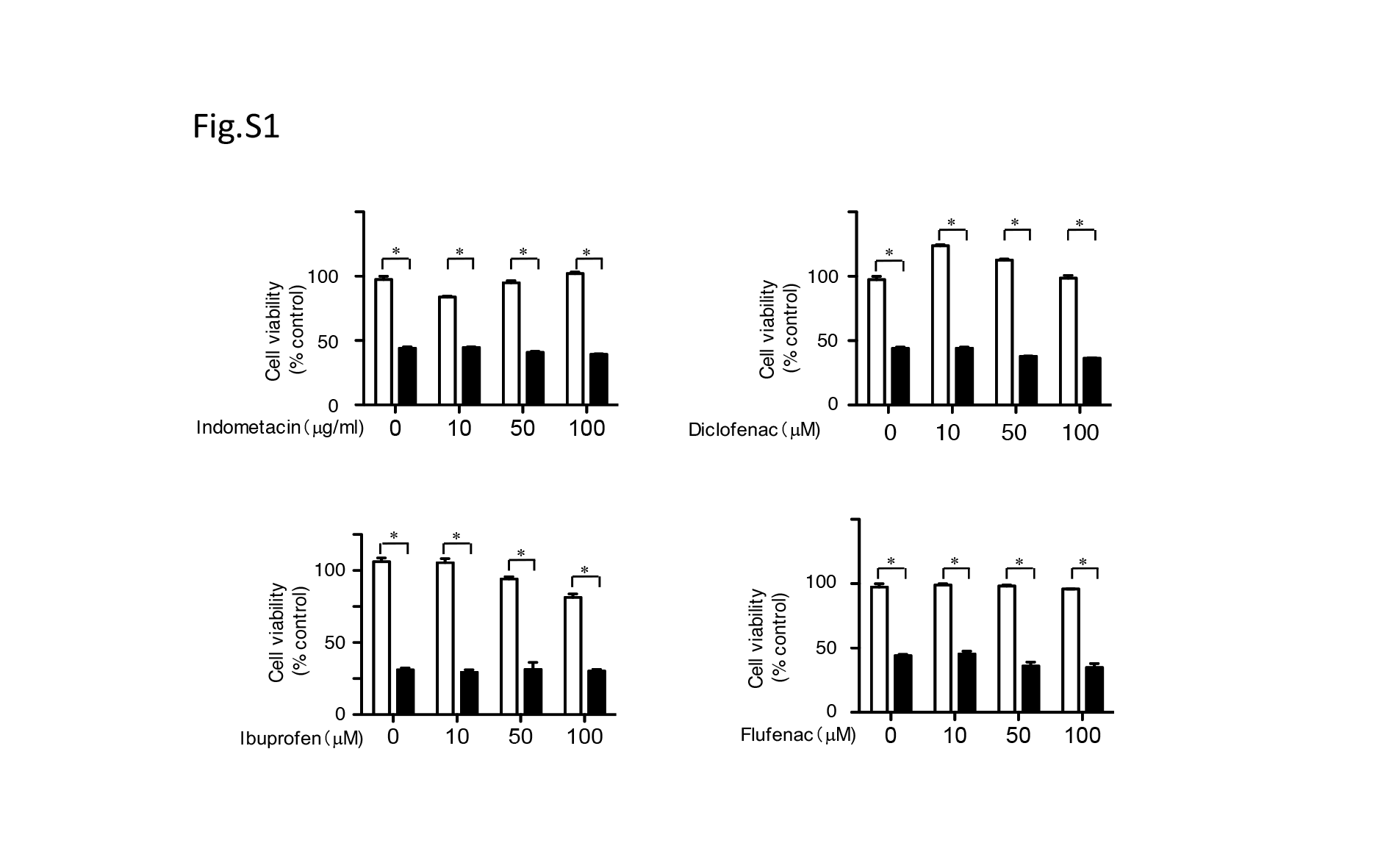

Supplement: Supplementary file 1 — Figure S1 [file 41420_2017_7_MOESM1_ESM.tif]
