# Supplementary material for: Mechanism of inhibition of Shiga-toxigenic Escherichia coli SubAB cytotoxicity by steroids and diacylglycerol analogues
Source: Cell Death Discov. 2018 Feb 14;4:22. doi: 10.1038/s41420-017-0007-4 (PMC5841432; doi:10.1038/s41420-017-0007-4)
Supplement: Supplementary file 2 — DECLARATION OF CONTRIBUTIONS TO ARTICLE [file 41420_2017_7_MOESM2_ESM.docx]

Supplementary Figure 1. NSAIDs did not inhibit SubAB-induced apoptosis.

HeLa cells were treated for 48 h with the indicated compounds in the presence or absence of mt or wt SubAB (0.2 μg/ml). Cell viability was determined with a Cell Counting Kit. Data are the means ± SD from three separate triplicate experiments. Student's t-test was utilized for comparisons with SubAB-treated cells without inhibitor. The symbol * represents p<0.01.
